# The Effect of a Multidisciplinary Lifestyle Intervention Program on Apelin-12, Vaspin and Resistin Concentrations in Children and Adolescents with Overweight and Obesity

**DOI:** 10.3390/nu16213646

**Published:** 2024-10-26

**Authors:** Sofia I. Karampatsou, George Paltoglou, Sofia M. Genitsaridi, Penio Kassari, Evangelia Charmandari

**Affiliations:** 1 Division of Endocrinology, Metabolism and Diabetes, First Department of Pediatrics, National and Kapodistrian University of Athens Medical School, ‘Aghia Sophia’ Children’s Hospital, 11527 Athens, Greece; sof.karampatsou@gmail.com (S.I.K.); gpaltoglou@gmail.com (G.P.); sgenitsaridi@gmail.com (S.M.G.); peniokassari@gmail.com (P.K.); 2 Department of Pediatrics, National and Kapodistrian University of Athens Nursing School, “P. and A. Kyriakou” Children’s Hospital, 11527 Athens, Greece; 3 Second Department of Pediatrics, National and Kapodistrian University of Athens Medical School, “P. and A. Kyriakou” Children’s Hospital, 11527 Athens, Greece; 4 Division of Endocrinology and Metabolism, Center for Clinical, Experimental Surgery and Translational Research, Biomedical Research Foundation of the Academy of Athens, 11527 Athens, Greece

**Keywords:** apelin-12, vaspin, resistin, overweight, obesity, childhood, adolescence, diet, sleep, exercise

## Abstract

**Background:** Obesity in childhood and adolescence has reached epidemic proportions in recent decades. **Methods:** In the present study, we determined the concentrations of apelin-12, vaspin and resistin in 106 children and adolescents with overweight or obesity before and after the implementation of a multidisciplinary, personalized lifestyle intervention program of diet, sleep and exercise for 1 year. All subjects attended our Center for the Prevention and Management of Overweight and Obesity in Childhood and Adolescence. **Results:** Following the lifestyle intervention, there were significant decreases in BMI (*p* < 0.01), apelin-12 (*p* < 0.05) and resistin (*p* < 0.01) concentrations, and an increase in vaspin (*p* < 0.01) concentration. Glucose was the best positive predictor of apelin-12 (b = 0.236, *p* < 0.05), and osteopontin was the best negative predictor of changes in apelin-12 (b = −0.299, *p* < 0.05). Vaspin correlated positively with adiponectin (b = 0.29, *p* < 0.05), while vitamin D (b = 0.621, *p* < 0.05) was the best positive predictor of vaspin. BMI z score (b = −0.794, *p* < 0.05), HDL (b = −0.284, *p* < 0.05) and HbA1C (b = −0.262, *p* < 0.05) were the best negative predictors of changes in vaspin. BMI z score was the best positive predictor of resistin (b = 0.437, *p* < 0.05). **Conclusions:** These findings suggest that apelin-12, vaspin and resistin correlate with indices of obesity, glucose, lipids and bone metabolism, while interaction with other proteins, such as osteopontin and adiponectin, was also noted. Therefore, apelin-12, vaspin and resistin may be used as biomarkers in children and adolescents with overweight and obesity.

## 1. Introduction

Obesity in childhood and adolescence has reached epidemic proportions in the last few decades. According to the World Health Organization (WHO), in 2019, 340 million children and adolescents aged 5–19 years had overweight or obesity [1]. In Europe, the highest rates are recorded in Mediterranean countries, with Greece having a leading role. Indeed, the prevalence of overweight and obesity in Greece ranges from 21% in preschool-aged children to 41% in school-aged children and adolescents and is significantly higher than in the rest of the European countries (15% and 25%, respectively) [2]. As a result, there is an urgent need for the prevention and management of childhood obesity. The investigation of the role of proteins that are secreted from the adipose tissue, i.e., adipokines, has attracted considerable interest in recent decades. Apelin, vaspin and resistin are adipokines that play a crucial role in the pathophysiology of obesity. However, little is known about their role in childhood and adolescence.

Apelin is a protein that plays an important role in various physiological processes, including cardiovascular function, fluid homeostasis and energy metabolism [3]. It was first described by Tatemoto et al. as an endogenous ligand of a G protein-coupled receptor named APJ in bovine stomach extracts [4]. It is encoded by the *APLN* gene and is expressed mainly in the adipose tissue, characterizing the protein as adipokine. In addition, it is expressed in the heart and the brain. The most well-described isoforms of apelin are apelin-13, -17 and -36, as well as the pyroglutaminated isoform of apelin-13 (Pyr(1)-apelin-13) [3,5]. Skeletal muscle is the major target tissue, where it mediates increased fuel consumption [3].

Apelin is involved in metabolic homeostasis. It improves insulin sensitivity, while insulin regulates apelin expression from the adipocytes [3,5]. Apelin concentrations are increased in patients with obesity, impaired glucose tolerance and diabetes mellitus type 2 (DM2). In addition, studies have demonstrated decreased apelin concentrations after weight loss due to diet, exercise or bariatric surgery, indicating a reverse regulation of apelin in obesity [3,5,6,7]. In children with obesity, apelin-12 was found to be a sensitive predictor of metabolic syndrome [8].

Vaspin is an adipokine that belongs to the serpina family, also named SERPINA12. It is so named because it is a serpin protein derived from the visceral adipose tissue [9]. It is encoded by the *SERPINA12* gene located on chromosome 14 (14q32.1) [10]. It is expressed mainly by the adipose tissue (visceral, subcutaneous and brown adipose tissue), as well as by the skin, liver, pancreas, placenta, stomach, cerebrospinal fluid, hypothalamus and ovaries [10].

As an adipokine, vaspin plays an important role in the pathogenesis of obesity and glucose homeostasis [9]. In the white adipose tissue (WAT) of rats, vaspin demonstrates insulin-sensitizing effects, with a negative effect on glucose concentrations [9]. In addition, vaspin acts in the hypothalamus of rats by reducing appetite and food intake, while rats fed a high-fat diet demonstrate increased vaspin concentrations [11]. In the pancreas, vaspin improves the function of β-cells, and the administration of vaspin ameliorates insulin sensitivity [12]. Increased vaspin concentrations are associated with an increased risk of obesity and metabolic syndrome [13,14]. As a result, vaspin could play a protective role in obesity because of its connection to insulin resistance via its insulin-sensitizing and anti-inflammatory effects [10,12]. Interestingly, polymorphisms of the vaspin gene influence body composition and the lipid profile in prepubertal healthy children [15].

Resistin was first described by Steppan et al. as a signaling molecule secreted by adipocytes [16]. Furthermore, it is secreted by peripheral blood mononuclear cells (PBMCs), macrophages and bone marrow cells and in smaller amounts from the pituitary gland, hypothalamus, epithelial cells of the gastrointestinal tract, adrenal glands, skeletal muscle and pancreas [17]. Resistin is the founding member of resistin-like molecules (RELMs) and acts in an endocrine, paracrine and autocrine fashion [17]. In mouse and rat adipose cells, resistin expression is upregulated by glucocorticoids, growth hormone, prolactin and testosterone and is suppressed by insulin and epinephrine [17]. The receptor of resistin has not been identified yet, although potential candidates include toll-like receptor 4 (TLR4), decorin (DCN), tyrosine kinase-like orphan receptor-1 (ROR-1), insulin growth factor-1 receptor (IGF-1R) and adenylyl cyclase-associated protein 1 (CAP1) [17].

Resistin induces inflammatory cytokines and promotes the expression of cell adhesion molecules [17], all taking place in a cycle wherein inflammation promotes the expression of resistin and resistin promotes inflammation [17]. As a result, resistin takes part in many situations that trigger inflammation, such as atherosclerosis and cardiovascular disease (CVD), non-alcoholic fatty liver disease (NAFLD), osteoporosis, cancer, Crohn’s disease, metabolic diseases, DM2 and autoimmune diseases [17,18].

Resistin is so named owing to its connection to resistance in insulin [16]. Its concentrations are higher in obese children than in children with normal weight [16,19,20]. Interestingly, resistin concentrations are decreased after administration of rosiglitazone, an anti-diabetic drug, as well as after Roux-en-Y gastric bypass (RYGB), adjustable gastric banding or intense exercise, while administration of recombined resistin reduces glucose tolerance [16,21,22].

The aim of our study was to determine the concentrations of the adipokines apelin-12, vaspin and resistin in children and adolescents with overweight and obesity before and after the implementation of a 1-year personalized multidisciplinary lifestyle intervention program of diet, sleep and physical activity, and to explore their associations with lipid and glucose metabolism.

## 2. Materials and Methods

### 2.1. Patients

One hundred and six (*n* = 106) children and adolescents were prospectively recruited to participate in our study. The subjects attended our Center for the Prevention and Management of Overweight and Obesity in Childhood and Adolescence, ‘Aghia Sophia’ Children’s Hospital, Athens, Greece, as described previously [23]. Subjects were excluded from the study if they had syndromic obesity [24]. Inclusion criteria were an age of 2–18 years, increased body mass index (BMI) [above the 85th percentile for age and gender according to International Obesity Task Force (IOTF) cut-off points] [25] and good compliance with the intervention program. More specifically, good compliance was determined during the appointments that the subjects attended throughout the duration of the study; adherence to the advice given by the pediatrician, pediatric endocrinologist, pediatric dietician and professional fitness personal trainer; and the decrease in BMI after the implementation of the program (mean decrease: 3.6 kg/m^2^) [23,26].

Subjects were classified as having obesity (*n* = 67.6%) and overweight (*n* = 32.4%) according to the IOTF cut-off points. The study was conducted in accordance with the Declaration of Helsinki and was approved by the Committee on the Ethics of Human Research of ‘Aghia Sophia’ Children’s Hospital (approval number: EB-PASCH-Mom: 28 November 2013, Re: 10290-14/05/2013 & Approval Number: EB-PASCH-MoM: 3 April 2018, Re: 7000-20/03/2018). All parents or guardians of the participants provided written informed consent, while assent was given by participants older than 7 years.

### 2.2. Methods

As previously described, the intervention was based on a personalized, comprehensive, multidisciplinary management lifestyle program [23]. Detailed medical history and clinical examination were performed by a single trained pediatrician. In all subjects, body weight and standing height were measured in light clothing and without shoes, using the same scale (Seca GmbH & Co. KG., Hamburg, Germany) and a Harpenden stadiometer (Holtain Limited, Crymych-Dyfed, UK), respectively. Waist circumference (WC) and hip circumference (HC) were measured using the same stretch-resistant tape (Seca GmbH & Co. KG., Hamburg, Germany), with the subject on standing position according to the WHO STEPS protocol [27]. Systolic blood pressure (SBP) and diastolic blood pressure (DBP) were determined twice, and the mean value was calculated by employing a sphygmomanometer (Comfort 20/40, Visomat, Parapharm, Metamorphosi, Attiki, Greece) using an age-appropriate cuff. The fat mass, muscle mass, bone mass, fat-free mass, total body water (TBW) and basal metabolic rate (BMR) of each participant were assessed via bioelectrical impedance analysis (BIA) (TANITA MC-780U Multi Frequency Segmental Body Composition Analyzer, Amsterdam, The Netherlands). Baseline hematologic, biochemical and endocrinologic investigations were conducted via blood sampling after a 12 h overnight fast at 08:00 h. Blood samples were centrifuged, and serum or plasma was separated immediately after collection and stored at −80 °C until assayed.

All participants entering the lifestyle intervention program, which provided personalized advice on healthy diet, sleep and physical exercise to patients and their families, were evaluated by a pediatrician, pediatric endocrinologist, pediatric dietician and professional fitness personal trainer. Participants were followed-up monthly if they had obesity and every two months if they had overweight. At the end of the study, after 1 year of lifestyle intervention, all participants were fully evaluated, and BIA and hematologic, biochemical and endocrinologic investigations were performed again.

The dietic assessment and intervention were based on the United States Department of Agriculture (USDA) “my plate” method [28]. The physical activity assessment and intervention started with an evaluation by a professional fitness personal trainer, who proposed a personalized physical activity plan. Our goal was to encourage exercise 3–4 times per week. In addition, we advised the patients and their families to have adequate and good-quality sleep depending on the age [29]. Finally, psychological assessment and intervention were offered by a pediatric clinical psychologist when needed in order to support patients and their families.

### 2.3. Assays

Standard hematologic and biochemical investigations were assayed via using an ADVIA 2110i analyzer (Roche Diagnostics, GmbH, Mannheim, Germany). Glycated hemoglobin A1c (HbA1c) was assayed via reversed-phase cation exchange High-Performance Liquid Chromatography (HPLC) on an automated HA-8160 glycohemoglobin analyzer (Arkray, Kyoto, Japan). Glucose, total cholesterol, triglycerides and High-Density Lipoprotein Cholesterol (HDL) concentrations were assayed via an ADVIA 1800 Siemens analyzer (Siemens Healthcare Diagnostics, Tarrytown, NY, USA). Apolipoproteins A1 (ApoA1), B (ApoB) and Lipoprotein (a) (Lp(a)) concentrations were assayed via latex particle-enhanced immunonephelometric assays on a BN ProSpec nephelometer (Dade Behring, Siemens Healthcare Diagnostics, Liederbach, Germany).

The concentrations of high-sensitivity C-reactive protein (hsCRP), cortisol, adrenocorticotropic hormone (ACTH), insulin-like growth factor-I (IGF-I) and insulin-like growth factor-binding protein 3 (IGFBP-3) were assayed via automated chemiluminescence immunoassays on an IMMULITE 2000 Immunoassay System (Siemens Healthcare Diagnostics Products Ltd., Camberley, Surrey, UK). Insulin concentrations were assayed via automated electrochemiluminescence immunoassays (ECLIA) (Analyzer Cobas e411, Roche Diagnostics, GmbH, Mannheim, Germany). Total 25-hydroxyvitamin D (25-OH-Vitamin D) was assayed via automated electrochemiluminescence immunoassay on a Modular Analytics E170 analyzer.

Apelin-12 concentrations were assayed via an enzyme-linked immunoassay (ELISA) kit (Cat. no. EK-057-23, Phoenix Pharmaceuticals, Burlingame, CA, USA; sensitivity: 0.07 ng/mL; intra-assay CV: <10% and inter-assay CV: <5%).

Vaspin concentrations were assayed via an ELISA kit (Cat. no. CSB-E09771h, Cusabio, Houston, TX, USA; sensitivity: 7.8 pg/mL; intra-assay CV: <8% and inter-assay CV: <10%).

Resistin concentrations were assayed via an ELISA kit (Cat. No. DRSN00; R&D Systems, Minneapolis, MN, USA; sensitivity: 0.055 ng/mL; intra-assay CV: 4.7% and inter-assay CV: 8.4%).

Adiponectin concentrations were assayed via an ELISA kit (Cat. No. BMS2032; eBioscience, ThermoFisher Scientific, Waltham, MA, USA; sensitivity: 0.01 ng/mL; intra-assay CV: 4.2% and inter-assay CV: 3.1%).

Leptin concentrations were assayed via an ELISA kit (Cat No. RD191001100; BioVendor, Heidelberg, Germany; sensitivity: 0.2 ng/mL; intra-assay CV: 5.9% and inter-assay CV: 5.5%).

Irisin concentrations were assayed via an ELISA kit (Phoenix Pharmaceuticals, Burlingame, CA, USA; Cat. no. EK-067-52; sensitivity: 4.15 ng/mL; intra-assay CV: <10% and inter-assay CV: <15%).

Fibroblast growth factor-21 (FGF-21) concentrations were assayed via an ELISA kit (Cat. No. DF2100; R and D Systems, Minneapolis, MN, USA; sensitivity: 8.69 pg/mL; intra-assay CV: 3.4% and inter-assay CV: 7.5%).

Fibroblast growth factor-23 (FGF-23) concentrations were assayed via an ELISA kit (Cat. No. 60-6600; Immutopics, San Clemente, CA, USA; sensitivity: 1.5 pg/mL; intra-assay CV: 3% and inter-assay CV: 6.2%).

Osteopontin concentrations were assayed via an ELISA kit (Cat No. DOST00; R&D Systems, Minneapolis, USA; sensitivity: 0.024 ng/mL; intra-assay CV: 3.1% and inter-assay CV: 5.9%).

Sclerostin concentrations were assayed via an ELISA kit (Cat. no. CSB-EL022416HU, Cusabio, Houston, TX, USA; sensitivity: 4.68 pg/mL; intra-assay CV: <10% and inter-assay CV: <12%).

Homeostasis model assessment (HOMA) to assess Insulin Resistance (IR) was calculated as follows: HOMA-IR = (fasting glucose [mg/dL] × fasting insulin [mU/L])/405. Tri-ponderal mass index (TMI) was calculated using the formula: TMI = mass divided by height cubed. Body mass index (BMI) and waist circumference z scores were based on the CDC Anthropometric Reference Data for Children and Adults [30].

### 2.4. Statistical Analysis

All assessed variables followed a normal distribution. All results are reported as mean ± standard error of the mean (SEM). Statistical significance was set at *p* < 0.05, while strong statistical significance (*p* < 0.01) is also noted. Regarding the effect size, when studying one group, it was 0.2498572, and when studying two groups, it was 0.1775627. Repeated-measures analysis of variance (ANOVA) tests were employed to compare all variables assessed at the time of initial assessment and at 12-month follow-up. Fischer’s (LSD) post hoc test was employed to reveal significant main effects. Pearson’s R coefficient was employed to evaluate potential correlations among the studied variables. Standard forward, stepwise multiple-regression models were employed to reveal potential predictors of the concentrations, and changes in the concentrations of apelin-12, vaspin and resistin were considered as dependent variables. In the first employed model, independent variables at the time of initial assessment were the anthropometric parameters (body weight, height, BMI, BMI z score, TMI, WC, HC, waist-to-hip ratio (WHR) and waist-to-height ratio (WHtR) measurements). In the second model, independent variables at the time of initial assessment were the body composition parameters (fat mass percentage, fat mass, muscle mass percentage, bone mass, fat-free mass, TBW and BMR). In the third model, independent variables at the time of initial assessment were the metabolic syndrome parameters (glucose concentration, SBP, WC, triglycerides and HDL) [30]. In the fourth model, independent variables at the time of initial assessment were the glucose metabolism and insulin sensitivity parameters (glucose, insulin, HbA1C and HOMA-IR measurements). In the fifth model, independent variables at the time of initial assessment were the adiposity parameters (adiponectin and leptin concentrations, WC z score, WHtR and fat mass). In the sixth model, independent variables at the time of initial assessment were the bone biochemical parameters (calcium, phosphorus, alkaline phosphatase (ALP), parathormone (PTH) and vitamin D).

Statistical analyses were performed using Statistica 8 software (StatSoft, Tulsa, OK, USA).

## 3. Results

### 3.1. Clinical Characteristics, Biochemical and Endocrinologic Parameters, Adipokines and Bone-Derived Proteins and Body Composition Parameters of All Subjects at Initial and Annual Assessment

A total of 106 children and adolescents were prospectively recruited to participate in our study for 1 year. Subjects were classified as obese (*n* = 71, 67.6%) or overweight (*n* = 34, 32.4%), of which 52.4% were males and 48.6% females; 44.8% were prepubertal, and 55.2% were pubertal subjects (Table 1).

The clinical characteristics (2A), biochemical parameters (2B), endocrinologic parameters (2C), adipokines and bone-derived proteins (2D) and body composition parameters (2E) of all subjects, as well as the respective statistically significant differences between initial and annual assessment, are presented in Table 2. Following one year of lifestyle interventions, there were significant decreases in BMI (*p* < 0.01), BMI z score (*p* < 0.01) and TMI (*p* < 0.01). More specifically, the percentage of subjects with obesity decreased from 67.6% to 33.3%, and the percentage of subjects with overweight increased from 32.4% to 36.2%, while at the annual assessment, 30.5% of subjects had normal BMI (Table 1). In addition, at the annual assessment, there were significant decreases in WC (*p* < 0.01), WC z-score (*p* < 0.01), WHR (*p* < 0.01) and WHtR (*p* < 0.01) (Table 2A).

Furthermore, following 1 year of the multidisciplinary personalized lifestyle intervention program, all subjects demonstrated significant decreases in hepatic enzymes (glutamic-oxaloacetic transaminase (SGOT) (*p* < 0.01), glutamic-pyruvic transaminase (SGPT) (*p* < 0.01) and gamma-glutamyl transferase (γ-GT) (*p* < 0.01)), insulin (*p* < 0.01), HbA1C (*p* < 0.01), HOMA-IR (*p* < 0.01), total cholesterol (*p* < 0.05), triglycerides (*p* < 0.05), low-density lipoprotein (LDL) (*p* < 0.01) and ApoB (*p* < 0.01) concentrations and significant increases in HDL (*p* < 0.01) and 25-OH vitamin D (*p* < 0.05) concentrations (Table 2B,C).

There were also significant increases in vaspin (*p* < 0.01) and sclerostin (*p* < 0.01) concentrations and decreases in apelin-12 (*p* < 0.01), resistin (*p* < 0.01), irisin (*p* < 0.01), osteopontin (*p* < 0.01), FGF-23 (*p* < 0.05) and leptin (*p* < 0.01) concentrations (Table 2D).

With respect to the body composition parameters, there were significant decreases in fat mass (*p* < 0.01) and fat mass percentage (*p* < 0.01) and increases in muscle mass percentage (*p* < 0.01), bone mass (*p* < 0.01), fat-free mass (*p* < 0.01) and TBW (*p* < 0.01) (Table 2E).

**Figure 1:**
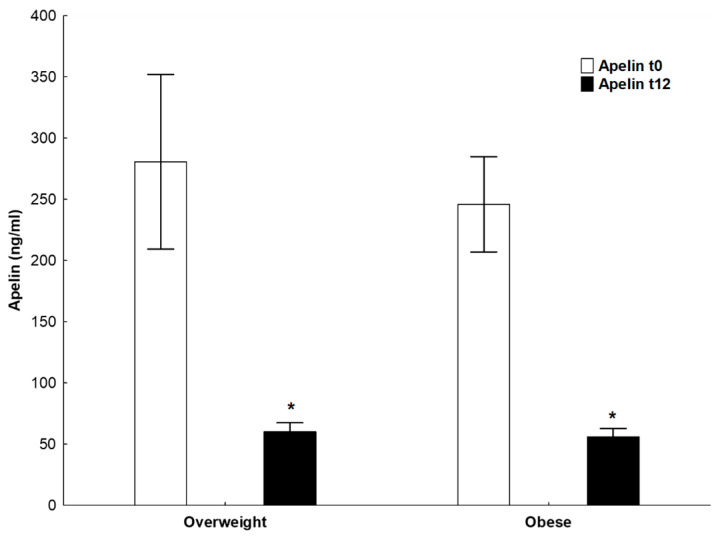
Bar plot showing apelin-12 concentrations of all subjects at initial assessment (white bars) and at annual (black bars) assessment. The bars represent the median and interquartile range. Asterisks indicate a statistically differences between the initial and annual assessments.

We further separately compared the alterations in adipokines between initial and annual assessment in subjects with obesity and those with overweight. As noted in Supplemental Table S1, at the annual assessment, subjects with obesity demonstrated significant increases in vaspin (*p* < 0.05) concentrations and significant decreases in apelin-12 (*p* < 0.01) concentrations (Figure 1) compared to the initial assessment. At the annual assessment, subjects with overweight presented with significant increases in vaspin (*p* < 0.01) concentrations and significant decreases in leptin (*p* < 0.01), apelin-12 (*p* < 0.05) (Figure 1) and resistin (*p* < 0.05) concentrations compared to the initial assessment.

### 3.2. Correlation Coefficient Analysis of All Subjects Categorized According to BMI and Pubertal Status

The correlation coefficient analysis of all subjects showed the following: Apelin-12 concentrations correlated negatively with cortisol (b = −0.92, *p* < 0.05). Vaspin concentrations correlated negatively with ApoB concentrations (b = −0.88, *p* < 0.05). Resistin concentrations correlated negatively with BMI (b = −0.93, *p* < 0.05), BMI z score (b = −0.89, *p* < 0.05), TMI (b = −0.91, *p* < 0.05), HC (b = −0.88, *p* < 0.05) and WHtR (b = −0.89, *p* < 0.05). Finally, changes in resistin correlated positively with WC (b = 0.93, *p* < 0.05), WC z score (b = 0.93, *p* < 0.05), WHtR (b = 0.90, *p* < 0.05), SBP (b = 0.89, *p* < 0.05), HbA1C (b = 0.91, *p* < 0.05), muscle mass percentage (b = 0.91, *p* < 0.05), fat-free mass (b = 0.91, *p* < 0.05) and TBW (b = 0.90, *p* < 0.05) and negatively with SGOT (b = −0.93, *p* < 0.05) (Table 3).

The correlation coefficient analysis of subjects with obesity showed the following: Apelin-12 concentrations correlated positively with osteopontin (b = 0.37, *p* < 0.05) concentrations. The change in apelin-12 concentrations correlated negatively with osteopontin (b = −0.32, *p* < 0.05) concentrations. Vaspin concentrations correlated positively with 25-OH vitamin D (b = 0.64, *p* < 0.05), adiponectin (b = 0.29, *p* < 0.05) and fat mass (b = 0.37, *p* < 0.05). The change in vaspin concentrations correlated negatively with age (b = −0.37, *p* < 0.05), body weight (b = −0.38, *p* < 0.05), height (b = −0.31, *p* < 0.05), BMI (b = −0.33, *p* < 0.05), fat mass (b = −0.42, *p* < 0.05), muscle mass (-0.33, *p* < 0.05), fat-free mass (b = −0.33, *p* < 0.05), bone mass (b = −0.33, *p* < 0.05), TBW (b = −0.34, *p* < 0.05), total cholesterol (b = −0.30, *p* < 0.05), ApoA (b = −0.40, *p* < 0.05), hsCRP (b = −0.43, *p* < 0.05), cortisol (b = −0.43, *p* < 0.05) and leptin (b = −0.39, *p* < 0.05) concentrations. Resistin concentrations correlated negatively with SGOT concentrations (b = −0.25, *p* < 0.05). Finally, the change in resistin concentrations correlated positively with ApoB (b = 0.28, *p* < 0.05) concentrations (Table 4).

The correlation coefficient analysis of subjects with overweight showed the following: Apelin-12 concentrations correlated positively with γGT (b = 0.45, *p* < 0.05) and FGF-21 (b = 0.55, *p* < 0.05) concentrations. The change in apelin-12 concentrations correlated negatively with cortisol (b = −0.50, *p* < 0.05) and FGF-21 (b = −0.50, *p* < 0.05) concentrations. Vaspin concentrations correlated positively with WHR (b = 0.54, *p* < 0.05) and glucose (b = 0.55, *p* < 0.05) concentrations and negatively with LDL (b = −0.53, *p* < 0.05) and albumin (b = −0.49, *p* < 0.05) concentrations. The change in vaspin concentrations correlated negatively with glucose concentrations (b = −0.54, *p* < 0.05) and HOMA-IR (b = −0.49, *p* < 0.05). Resistin concentrations correlated positively with fat mass (b = 0.40, *p* < 0.05), leptin (b = 0.40, *p* < 0.05) and sclerostin (b = 0.54, *p* < 0.05) concentrations and negatively with vitamin D (b = −0.34, *p* < 0.05) (Table 5).

The correlation coefficient analysis in prepubertal subjects showed the following: Apelin-12 concentrations correlated positively with cortisol (b = 0.51, *p* < 0.05) and FGF-21 (b = 0.76, *p* < 0.05) concentrations. The change in apelin-12 correlated negatively with cortisol (b = −0.37, *p* < 0.05) and FGF-21 (b = −0.74, *p* < 0.05) concentrations. Vaspin concentrations correlated positively with 25-OH vitamin D concentrations (b = 0.78, *p* < 0.05). The change in vaspin concentrations correlated positively with TMI (b = 0.40, *p* < 0.05) and LDL concentrations (b = 0.37, *p* < 0.05). Resistin concentrations correlated positively with BMI (b = 0.29, *p* < 0.05), irisin (b = 0.47, *p* < 0.05) and FGF-23 (b = 0.30, *p* < 0.05) concentrations. Finally, the change in resistin correlated negatively with irisin (b = −0.34, *p* < 0.05) concentrations (Table 6).

The correlation coefficient analysis in pubertal subjects showed the following: Apelin-12 concentrations correlated positively with calcium (b = 0.33, *p* < 0.05), glucose (b = 0.43, *p* < 0.05) and osteopontin (b = 0.40, *p* < 0.05) concentrations and negatively with cortisol concentrations (b = −0.33, *p* < 0.05). The change in apelin-12 correlated negatively with osteopontin concentrations (b = −0.35, *p* < 0.05). Vaspin concentrations correlated positively with hsCRP (b = 0.34, *p* < 0.05), total cholesterol (b = 0.34, *p* < 0.05), HDL (b = 0.33, *p* < 0.05), apoA1 (b = 0.33, *p* < 0.05) and cortisol (b = 0.42, *p* < 0.05) concentrations, while the change in vaspin concentrations correlated negatively with total cholesterol (b = −0.40, *p* < 0.05), HDL (b = −0.36, *p* < 0.05), ApoA1 (b = −0.39, *p* < 0.05), cortisol (b = −0.46, *p* < 0.05) and hsCRP (b = −0.42, *p* < 0.05) concentrations. Resistin concentrations correlated negatively with glucose (b = −0.33, *p* < 0.05) and 25-OH vitamin D (b = −0.31, *p* < 0.05) concentrations. Finally, the change in resistin concentrations correlated positively with glucose concentrations (b = 0.31, *p* < 0.05) (Table 7).

### 3.3. Multivariate Linear Regression Analysis of Anthropometric, Body Composition, Metabolic Syndrome, Glucose Metabolism, Adiposity and Bone Metabolism Parameters

**Figure 2:**
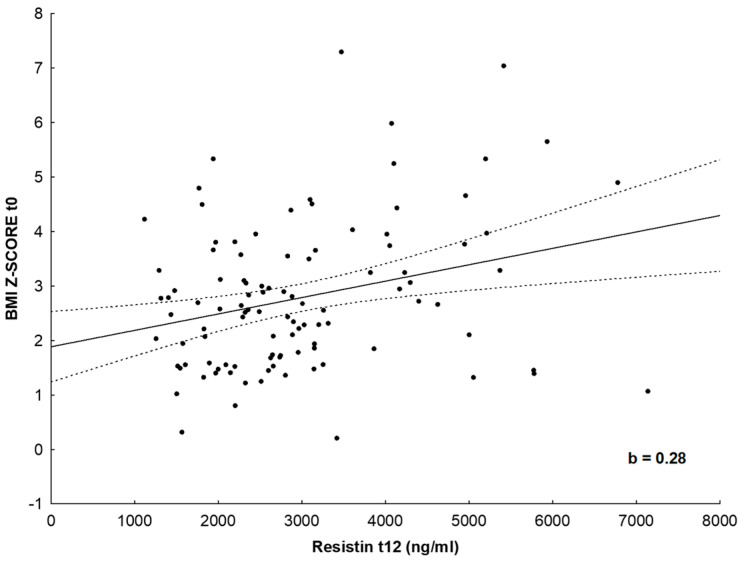
Scatter plot showing standard forward, stepwise multiple linear regression model. BMI z score was the best positive predictor of resistin concentrations after the implementation of the life-style intervention program for 1 year. The dots represent individual patients.

In the multivariate linear regression analysis, when anthropometric parameters (body weight, height, BMI, BMI z score, TMI, WC, HC, WHR and WHtR) at initial assessment were taken as independent variables in a standard, forward stepwise regression model, BMI z score (b = 0.437, *p* < 0.05) was the best positive predictor of resistin concentrations after the implementation of the lifestyle intervention program for 1 year (dependent variable) (Figure 2). In addition, height (b = 2.401, *p* < 0.05) and TMI (b = 2.132, *p* < 0.05) were the best positive predictors, while body weight (b = -2.556, *p* < 0.05) and BMI z score (b = −0.794, *p* < 0.05) were the best negative predictors of the change in vaspin concentrations (Supplemental Table S2).

When body composition parameters (fat mass percentage, fat mass, muscle mass percentage, bone mass, fat free mass, TBW and BMR) at initial assessment were taken as independent variables in a standard, forward stepwise regression model, no predictor was found.

When metabolic syndrome parameters at initial assessment (glucose concentration, SBP, WC, triglycerides and HDL concentrations) were taken as independent variables in a standard, forward stepwise regression model, glucose concentration was the best positive predictor (b = 0.236, *p* < 0.05) of apelin-12 concentrations at the annual assessment (dependent variable), while glucose concentration (b = −0.281, *p* < 0.05) and HDL (b = −0.284, *p* < 0.05) were the best negative predictors of changes in vaspin concentrations (Supplemental Table S3).

**Figure 3:**
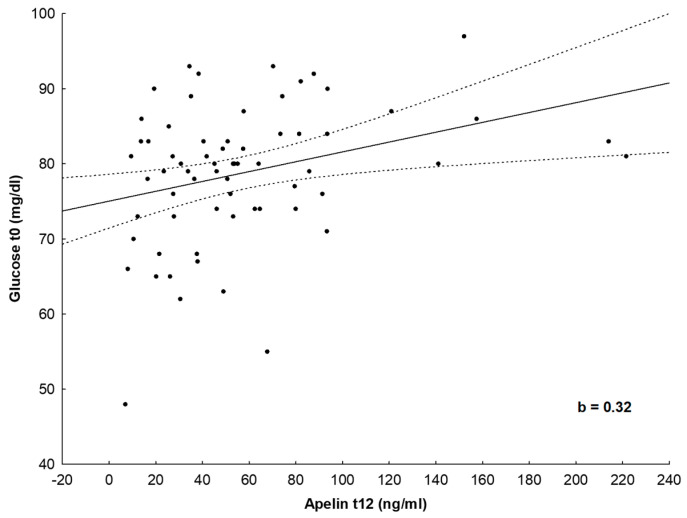
Scatter plot showing standard, forward, stepwise multiple linear regression model. Glucose concentrations were the best positive predictor of apelin-12 after the implementation of the lifestyle intervention program for 1 year. The dots represent individual patients.

When glucose metabolism parameters (glucose, insulin, HbA1C and HOMA-IR) at initial assessment were taken as independent variables in a standard, forward stepwise regression model, glucose concentration was the best positive predictor (b = 0.328, *p* < 0.05) of apelin-12 at the annual assessment after 1 year of the implementation of the lifestyle intervention program (dependent variable) (Figure 3), and HbA1C concentration was the best negative predictor (b = −0.262, *p* < 0.05) of changes in vaspin (dependent variable) (Supplemental Table S4).

When adiposity parameters (adiponectin and leptin concentrations, WC, WC z score, WHtR and fat mass) at initial assessment were taken as independent variables in a standard, forward stepwise regression model, WC z score was the best negative predictor (b = −0.671, *p* < 0.05) of changes in apelin-12 (dependent variable) (Supplemental Table S5).

When bone metabolism parameters (calcium, phosphorus, ALP, PTH, 25-OH vitamin D, osteopontin and FGF-23) at initial assessment were taken as independent variables in a standard, forward stepwise regression model, osteopontin was the best negative predictor (b = −0.299, *p* < 0.05) of changes in apelin-12 (dependent variable), and 25-OH vitamin D concentrations were the best positive predictors (b = 0.621, *p* < 0.05) of vaspin concentrations at annual assessment after the implementation of the lifestyle intervention program for 1 year (dependent variable) (Supplemental Table S6).

## 4. Discussion

In our study, we determined serum apelin-12, vaspin and resistin concentrations in children and adolescents with overweight and obesity before and after the implementation of a 1-year multidisciplinary, personalized lifestyle intervention program including a healthy diet, good-quality sleep and regular exercise. We demonstrated that the implementation of this lifestyle intervention program was successful, resulting in significant decreases in BMI and BMI z scores, as well as significant decreases in apelin-12 and resistin concentrations and significant increases in vaspin concentrations. In addition, there was significant improvement in cardiometabolic risk factors, as indicated by the improvement in anthropometric parameters (decreases in WHR, WHtR, WC z score and fat percentage and increases in muscle mass and fat-free mass), the lipid profile (decreases in total cholesterol, LDL and ApoB and increases in HDL concentrations) and glucose metabolism (decreases in insulin HbA1C and HOMA-IR). Furthermore, we noted associations of apelin-12, vaspin and resistin with adipose tissue, glucose metabolism, lipid metabolism and bone metabolism. To the best of our knowledge, this is the first study in children and adolescents that demonstrates an association of apelin-12, vaspin and resistin concentrations with obesity and cardiometabolic risk factors following a 1-year lifestyle intervention program.

Apelin-12 is an adipokine expressed by the adipose tissue that plays an important role in metabolic homeostasis. It ameliorates insulin sensitivity and is increased in patients with obesity and DM2 [3,5]. In our study, we demonstrated that apelin-12 decreased after the implementation of the 1-year lifestyle intervention program, which resulted in a decrease in the BMI, while the WC z score was the best negative predictor of changes in apelin-12. Our findings concur with previous studies that demonstrated increased apelin-12 concentrations in girls with obesity [7] and decreased apelin-12 concentrations in underweight children [31] compared with a control group. In addition, in females with obesity, apelin-12 concentrations decreased after 2 months of aerobic and resistance exercise [6]. On the other hand, there are studies that demonstrated negative correlations of apelin with HOMA indices and insulin concentrations, while apelin concentrations were lower in subjects with obesity, although the sample size was very small [32,33]. Moreover, apelin demonstrates a positive correlation with indices of obesity such as BMI and WC [34,35]. WC is a surrogate marker of obesity and fat mass [36]. These results demonstrate the association of apelin-12 with adipose tissue, although more studies are needed to investigate the role of apelin-12 as a biomarker of obesity.

As for glucose homeostasis, in our study, we noted a positive correlation of apelin-12 concentrations with glucose concentrations in adolescents, and glucose concentrations were the best positive predictor of apelin-12 concentrations. Similarly to our results, in a study of children with obesity, apelin-12 concentrations correlated positively with indices of glucose homeostasis, such as glucose, insulin and HOMA-IR [7], indicating that apelin overexpression might have a protective role against insulin resistance [3]. These findings highlight the potential role of apelin-12 as a biomarker of insulin resistance, especially in adolescents.

Apelin is an adipokine that is secreted from the liver [37]. In our study, we demonstrated a positive correlation of apelin-12 concentration with γ-GT in subjects with overweight. In addition, there was a positive correlation of apelin-12 with FGF-21 concentrations and a negative correlation of the change in apelin-12 with FGF-21 concentrations in subjects with overweight and in prepubertal subjects. FGF-21 is a protein secreted by the liver that plays a crucial role in glucose and lipid metabolism [38,39]. Moreover, apelin-36 has been found to be increased in adults with NAFLD compared with a control group [34,40]. Interestingly, in a study of obese mice, apelin treatment resulted in decreased hepatic steatosis by reducing de novo lipogenesis, although this may be an indirect effect due to the increase in insulin sensitivity [41]. An interesting question that arises is the potential role of apelin as a biomarker in NAFLD in the pediatric population. To the best of our knowledge, this is the first study to describe a correlation between adipokine apelin-12 and hepatokine FGF-21, implicating the connection between adipose tissue and the liver, serving as a prompt for further research.

Osteopontin is a protein secreted from bone, playing a crucial role in bone size and density [42]. In our study, apelin-12 correlated positively with calcium in adolescents, while osteopontin correlated positively with apelin-12 and negatively with the change in apelin-12 in subjects with obesity and adolescents. In addition, osteopontin was the best negative predictor of the change in apelin-12. Interestingly, osteopontin contributes to chronic low-grade inflammation in obesity [43] and is up-regulated in obesity and insulin resistance [26,44]. Our results are in agreement with an in vitro study of vascular smooth muscle cells, which showed that apelin up-regulated osteopontin secretion through growth response factor-1 (Egr-1) [45]. The above findings indicate crosstalk between bone and adipose tissue in adolescents, given that apelin-12 and osteopontin could take part in the same pathways as obesity.

Vaspin is a protein secreted by adipose tissue; as a result, it is included in the adipokines family, with its main role being to improve insulin sensitivity [9,12]. In our study, vaspin concentrations increased after the implementation of a lifestyle intervention program and the corresponding decrease in BMI. Vaspin correlated with measurements of obesity. More specifically, vaspin concentrations correlated positively with fat mass in subjects with obesity and WHR in subjects with overweight. In addition, the change in vaspin correlated positively with TMI and negatively with body weight, BMI, muscle mass and fat-free mass in subjects with obesity. Interestingly, TMI and height were the best positive predictors, while BMI z score and body weight were the best negative predictors of changes in vaspin concentrations. Similar to our findings, previous studies have demonstrated a positive correlation between vaspin and WC in adults with obesity [34]. In studies of children with obesity and/or metabolic syndrome, vaspin concentrations were found to be lower than those in the control group [46] and correlated positively with body weight and BMI [13,14]. Interestingly, the administration of vaspin to rats reduces appetite [11] and body weight [47], while specific vaspin gene polymorphisms are associated with BMI variations in prepubertal children [15]. We speculate that vaspin is increased in obesity as a compensatory mechanism to decrease insulin resistance and improve metabolism by improving β pancreatic cell function [12].

Vaspin is an adipokine that is characterized by its insulin-sensitizing effects [9]. In our study, vaspin concentrations correlated positively with glucose concentrations, while changes in vaspin correlated negatively with glucose concentrations and HOMA-IR. Moreover, HbA1C and glucose concentrations were the best negative predictors of changes in vaspin concentrations. These results are in accordance with several previous studies. In adults, vaspin was negatively affected by insulin concentrations [48]. In animal studies, the administration of metformin, an anti-diabetic agent, resulted in increased vaspin secretion [49], while vaspin decreased glucose concentrations and improved insulin sensitivity in rats fed a high-fat diet [12]. These findings indicate a potential role of vaspin as a biomarker not only of insulin resistance but also of metabolic syndrome in children and adolescents living with obesity.

As for the lipid profile, in our study, vaspin correlated negatively with ApoB and LDL and positively with total cholesterol, HDL and ApoA1, while changes in vaspin correlated negatively with total cholesterol and ApoA1 and negatively with HDL. HDL concentrations were the best negative predictor of changes in vaspin concentrations. In previous studies of children with obesity and/or metabolic syndrome, vaspin also correlated positively with triglycerides and HDL [14,46]. Interestingly, in a study of postmenopausal women, vaspin demonstrated a positive correlation with pulse-wave velocity, a marker of arterial stiffness and, hence, cardiovascular disease [50]. Therefore, vaspin is a potential biomarker of dyslipidemia in children and adolescents with obesity.

In our study, vaspin concentrations correlated positively with 25-OH vitamin D concentrations in subjects with obesity and adolescents, while 25-OH vitamin D concentrations were the best positive predictor of vaspin. Other studies also have shown similar results. In a study of healthy women, 25-OH vitamin D and vaspin concentrations demonstrated a positive correlation [51]. We also demonstrated that the change in vaspin concentrations correlated negatively with bone mass in subjects with obesity. Our findings concur with those of previous studies, demonstrating a positive correlation between vaspin and bone mineral density [50]. Moreover, in a study of rats fed a high-fat diet, the administration of vaspin resulted in the restoration of the impacted bone strength by promoting osteoblastic differentiation [47]. This relation may indicate crosstalk between adipose and bone tissue and that vaspin may play a role in osteoporosis treatment.

As for the connection of vaspin with other adipokines, vaspin concentrations correlated positively with adiponectin concentrations in subjects with obesity, and changes in vaspin correlated negatively with leptin concentrations. Our results concur with those of other studies that demonstrated a positive correlation between vaspin and adiponectin in rats [52]. Vaspin exerts insulin-sensitizing and anti-inflammatory actions [10,12] similar to those of adiponectin [53]. In animal studies, there was a positive correlation between leptin and vaspin concentrations [50]. In addition, leptin administration increased mRNA vaspin expression from WAT after fasting, indicating that leptin could play a role in vaspin secretion [49]. It is not known yet if the above-mentioned adipokines directly interact with each other or take part in the same pathophysiologic mechanisms of obesity and insulin resistance.

Resistin is an adipokine that plays an important role in the pathophysiology of insulin resistance and obesity [16]. In our study, resistin concentrations decreased after the implementation of a lifestyle intervention program and decreases in BMI. In addition, resistin correlated positively with indices of obesity, such as BMI, BMI z-score, TMI, HC, WHtR and leptin, while changes in resistin concentrations correlated positively with WC, WC z score, WHtR, muscle mass percentage and fat-free mass. Interestingly, BMI z score was the best positive predictor of resistin concentrations. Our results concur with those of several previous studies. More specifically, in studies of young, non-diabetic adults and children with obesity, resistin levels were higher than those of a control group [54,55]. Moreover, after the implementation of a 1.5-year program with diet and exercise, the change in serum resistin correlated positively with changes in BMI, body fat, fat mass, visceral fat, and glucose and insulin concentrations [54]. Along the same lines, in adults with obesity, resistin decreased after Roux-en-Y gastric bypass (RYGB) or adjustable gastric banding, as well as in adolescents with overweight or obesity after 8 months of intensive exercise [21,22]. Resistin was also positively correlated with BMI, ΒΜΙ z score and leptin in obese and non-obese children and adults [16,19,20,56,57]. These results indicate an important role of resistin in the pathophysiology of obesity, which may be used as a biomarker of obesity in children and adolescents.

As for glucose metabolism, in our study, resistin correlated negatively with glucose concentrations in adolescents, while changes in resistin correlated positively with glucose in prepubertal subjects and with HbA1C in all subjects. In other studies of children or adults with DM2, resistin showed positive correlations with HOMA-IR, HbA1C and C-peptide and was increased in children with DM1 when compared to controls [56,58,59]. Interestingly, resistin concentrations decreased after the administration of rosiglitazone, an anti-diabetic drug, while the administration of recombinant resistin reduced glucose tolerance [16]. The above-mentioned results may explain the important role of resistin in glucose metabolism and insulin resistance.

As for the lipid profile, in our study, changes in resistin concentrations correlated positively with ApoB concentrations in subjects with obesity. Jones et al. demonstrated that after 8 months of intensive exercise in adolescents with obesity, the change of resistin correlated positively with changes in triglyceride concentrations [22]. Interestingly, in children with obesity with or without NAFLD, resistin demonstrated a positive correlation with HDL [56]. More studies are needed in order to investigate the impact of resistin on lipid metabolism, especially in childhood and adolescence.

Vitamin D takes part in glucose homeostasis and obesity, with actions characterized as autocrine, paracrine and endocrine [60]. In our study, resistin concentrations correlated negatively with 25-OH vitamin D in subjects with overweight and in adolescents. Similarly to our findings, resistin was found to have a negative correlation with vitamin D, while vitamin D was an independent predictor of resistin concentrations in postmenopausal women and in children [58,61,62]. In addition, patients with metabolic syndrome and vitamin D insufficiency demonstrate increased resistin concentrations [62].

In our study, resistin correlated negatively with SGOT, an enzyme secreted mainly by the liver. Our results concur with those of other studies. More specifically, in a study of children with obesity with and without NAFLD, resistin correlated with SGOT, SGPT and γ-GT. Furthermore, resistin can differentiate children with obesity and with or without hepatopathy [56]. In a study of patients with chronic hepatitis B infection, resistin correlated with SGPT but not with SGOT [63]. Furthermore, resistin was positively correlated to the severity of fibrosis in adults with NAFLD [57,64]. More studies are needed in order to evaluate the association of resistin with NAFLD.

Our study has several strengths. Firstly, the lifestyle intervention program was implemented by a large, multidisciplinary team of pediatricians, pediatric endocrinologists, a pediatric dietician and a professional fitness personal trainer in a specialized, tertiary referral ‘Center for the Prevention and Management of Overweight and Obesity in Childhood and Adolescence’. As a result, the intervention was delivered by experts in the field of childhood obesity. In addition, the population sample included subjects from both BMI categories (overweight and obesity) and pubertal statuses (prepubertal and pubertal).

Our study has some limitations. Firstly, we did not address potential confounding factors broadly in our data analysis (pubertal status, gender and baseline metabolic conditions), although we categorized the subjects according to pubertal status and BMI in the correlation coefficient analysis. In addition, we did not use a control group in the statistical analysis because all the subjects were patients attending our Center for the Prevention and Management of Overweight and Obesity in Childhood and Adolescence. Therefore, all patients were either self-referred or referred to our center by their general pediatricians or general practitioners to help them reduce their weight. As a result, they all received the multidisciplinary assessment and management regardless of their BMI. Furthermore, the design of the study was such that we aimed to evaluate our cohort before and after the intervention; as a result, all participants served as their own controls.

In our study, we provide original and useful information that may lay the foundation for future studies. Firstly, it would be useful to study a population of children and adolescents with obesity for a period after the end of the intervention and investigate if the concentrations of apelin-12, vaspin and resistin, as well as the BMI, would remain stable. This could provide the strongest evidence of the role of these proteins as biomarkers of obesity in childhood and adolescents. Secondly, there are no normal values for apelin-12, vaspin and resistin in children and adolescents. Therefore, it would be extremely interesting to study if our results are above or lower than what is expected in a population with normal BMI. Lastly, future studies are required to compare different types of intervention programs, such as healthy diet versus exercise, in order to investigate the impact of each intervention on the studied proteins.

Biomarkers are proteins that can be very helpful in the diagnosis and treatment of obesity. In our study, we demonstrated that the concentrations of apelin-12, vaspin and resistin change with alterations of anthropometric parameters, glucose and lipid metabolism indices and reductions in BMI. Therefore, measuring apelin-12, vaspin and resistin before and after an intervention program could be useful in evaluating its success.

## 5. Conclusions

In conclusion, a multidisciplinary personalized lifestyle intervention program including a healthy diet, good-quality sleep and regular exercise is effective in reducing BMI and improving the cardiometabolic profile. Although it is not clear which aspect of the intervention played the most important role in decreasing the BMI, we believe that it is the combination of all elements of the intervention (diet, sleep and exercise) that resulted in the reduction in BMI. In addition, we showed that apelin-12, vaspin and resistin correlated with indices of obesity, glucose and lipid metabolism. Furthermore, apelin-12, vaspin and resistin were associated with other adipokines and bone-derived proteins, such as FGF-21, FGF-23, irisin, osteopontin, sclerostin, adiponectin and leptin.

Taken together, our findings suggest that apelin-12, vaspin and resistin may be used as biomarkers in children and adolescents with overweight and obesity. More studies are needed in order to investigate the underlying pathophysiological mechanisms.

## Figures and Tables

**Table 1:** Gender, pubertal status and BMI category of all subjects at initial and annual assessment.

	Initial Assessment	Annual Assessment
Gender		
Male	54 (52.4%)	
Female	51 (48.6%)	
Pubertal status		
Prepubertal	47 (44.8%)	28 (26.7%)
Pubertal	58 (55.2%)	77 (73.3%)
BMI category		
Obese	71 (67.6%)	35 (33.3%)
Overweight	34 (32.4%)	38 (36.2%)
Normal BMI	-	32 (30.5%)

Abbreviations: BMI, body mass index; categorical variables are presented as frequencies (percentages).

**Table 2:** Clinical characteristics (A), biochemical parameters (B), endocrinologic parameters (C), adipokines and bone-derived proteins (D), and body composition parameters (E) in all subjects at initial and annual assessment.

**(A)** **Clinical characteristics**	**Initial Assessment**	**Annual Assessment**	***p*** **Value**
Age (years)	10.56 (±0.31)	11.64 (±0.31)	<0.01 **
BW (kg)	63.25 (±2.31)	61.95 (±2.31)	NS
Height (cm)	147.05 (±1.83)	152.36 (±1.96)	<0.01 **
BMI (kg/m^2^)	27.98 (±0.49)	25.03 (±0.46)	<0.01 **
BMI z score	2.78 (±0.13)	1.72 (±0.11)	<0.01 **
TMI (kg/m^2^)	19.07 (±0.25)	16.59 (±0.33)	<0.01 **
SBP (mmHg)	113.98 (±1.32)	113.13 (±1.33)	NS
DBP (mmHg)	66.24 (±1.16)	67.3 (±1.01)	NS
WC (cm)	87.56 (±1.55)	84.22 (±1.52)	<0.01 **
WC z-score	1.31 (±0.09)	0.89 (±0.09)	<0.01 **
HP (cm)	92.24 (±1.73)	93.16 (±1.64)	NS
WHR	0.96 (± 0.01)	0.90 (±0.01)	<0.01 **
WHtR	0.59 (±0.01)	0.56 (±0.02)	<0.01 **
**(B)** **Biochemical parameters**	**Initial Assessment**	**Annual Assessment**	***p*** **Value**
hsCRP (mg/L)	0.36 ± 0.09	0.15 ± 0.01	<0.05 *
Glucose (mg/dL)	79.25 (±0.84)	79.32 (±1.08)	NS
HbA1C (%)	5.27 (±0.03)	5.21 (±0.02)	<0.01 **
HOMA-IR	3.27 (±0.23)	2.57 (±0.17)	<0.01 **
Urea (mg/dL)	28.94 (±0.61)	27.24 (±0.63)	NS
Creatinine (mg/dL)	0.48 (±0.01)	0.54 (±0.02)	<0.01 **
SGOT (U/L)	24.4 (±0.68)	21.05 (±0.64)	<0.01 **
SGPT (U/L)	22.61 (±1.34)	17.22 (±0.67)	<0.01 **
γGT (U/L)	14.99 (±0.59)	12.44 (±0.49)	<0.01 **
Albumin (g/dL)	4.63 (±0.04)	4.6 (±0.04)	NS
Calcium (mmol/L)	9.97 (±0.04)	9.78 (±0.03)	<0.01 **
Total cholesterol (mg/dL)	155.97 (±3.01)	148 (±3.67)	<0.05 *
Triglycerides (mg/dL)	85.45 (±4.6)	78.76 (4.11)	<0.05 *
HDL (mg/dL)	49.83 (±1.52)	54.35 (±1.38)	<0.01 **
LDL (mg/dL)	91.76 (±2.08)	83.02 (±2.17)	<0.01 **
ApoA1 (mg/dL)	141.61 (±2.29)	141.81 (±2.53)	NS
ApoB (mg/dL)	74.68 (±1.93)	68.68 (±1.49)	<0.01 **
Lp(a) (mg/dL)	15.75 (±2.35)	17.15 (±2.51)	NS
**(C)** **Endocrinologic parameters**	**Initial Assessment**	**Annual Assessment**	***p*** **Value**
IGF-I (ng/mL)	331.22 (±18.07)	448.02 (±22.02)	<0.01 **
IGFBP-3 (μg/mL)	5.21 (±0.1)	5.46 (±0.1)	<0.01 **
Insulin (μUI/mL)	16.89 (±1.06)	13.51 (±0.81)	<0.01 **
PTH (pg/mL)	34.69 (±1.17)	37.47 (±1.13)	<0.05 *
25-OH-Vitamin D (ng/mL)	22.01 (±0.98)	25.16 (±1)	<0.05 *
ACTH (pg/mL)	29.2 (±1.78)	25.91 (±1.65)	<0.01 **
Cortisol (μg/dL)	15.12 (±1.07)	13.33 (±0.61)	NS
**(D)** **Adipokines and bone-derived proteins**	**Initial Assessment**	**Annual Assessment**	***p*** **Value**
Apelin-12 (ng/mL)	255.9 (±34.22)	57.18 (±5.34)	<0.01 **
Vaspin (pg/mL)	0.23 (±0.07)	0.30 (±0.07)	<0.01 **
Resistin (μg/mL)	3.75 (±0.18)	2.98 (±0.12)	<0.01 **
Adiponectin (μg/mL)	22.52 (±1.78)	22.55 (±1.75)	NS
Leptin (ng/mL)	30.63 (±2.37)	20.68 (±1.61)	<0.01 **
Irisin (μg/mL)	0.45 (±0.03)	0.28 (±0.02)	<0.01 **
FGF-21 (pg/mL)	39.02 (±4.46)	36.09 (±4)	NS
FGF-23 (pg/mL)	13.47 (±4.28)	10.52 (±3.88)	<0.05 *
Osteopontin (ng/mL)	29.11 (±2.26)	21.37 (±1.48)	<0.01 **
Sclerostin (pg/mL)	1.97 (±0.39)	4.81 (±0.58)	<0.01 **
**(E)** **Body composition parameters**	**Initial Assessment**	**Annual Assessment**	***p*** **Value**
Fat Percentage (%)	37 (±0.66)	31.23 (±0.63)	<0.01 **
Fat mass (kg)	24.9 (±1.28)	19.95 (±1.03)	<0.01 **
Muscle mass percentage (%)	38.59 (±1.24)	39.84 (±1.24)	<0.01 **
Bone Mass (kg)	2.09 (±0.06)	2.15 (±0.06)	<0.01 **
Fat-free mass (kg)	40.68 (±1.31)	41.99 (±1.3)	<0.01 **
TBW (kg)	29.8 (±0.96)	30.76 (±0.96)	<0.01 **
BMR (Kilojoule)	6663 (±145)	6678 (±138)	NS

Abbreviations: ACTH, adrenocorticotropic hormone; ApoA1, apolipoprotein A1; ApoB, apolipoprotein B; BMI, body mass index; BMR, basal metabolic rate; BW, body weight; DBP, diastolic blood pressure; FGF, fibroblast growth factor; γGT, gamma-glutamyl transferase; HbA1C, hemoglobin A1C; HDL, high-density lipoprotein; HOMA-IR, homeostatic model assessment for insulin resistance; HP, hip circumference; hsCRP, high-sensitivity C-reactive protein; IGF1, insulin-like growth factor 1; IGF-BP3, IGF-binding protein 3; LDL, low-density lipoprotein; Lp(a), lipoprotein a; PTH, parathormone; SBP, systolic blood pressure; SGOT, serum glutamic-oxaloacetic transaminase; SGPT, glutamic-pyruvic transaminase; TBW, total body water; TMI, tri-ponderal mass index; 25-OH-Vitamin D, total 25-OH vitamin D; WC, waist circumference; WHR, waist-to-hip ratio; WHtR, waist-to-height ratio. All variables are presented as mean ± SE of the mean. All measured variables were compared by employing a one-way ANOVA. Significant main effects were revealed by the LSD post hoc test. Statistical significance was set at *p* < 0.05, as shown in bold and indicated by an asterisk, while strong significance was set at *p* < 0.01, as shown in bold and indicated by two asterisks. NS: nonsignificant (*p* > 0.05) difference.

**Table 3:** Correlation coefficients of the apelin-12, vaspin and resistin concentrations in all subjects at initial assessment.

	Apelin-12	Change in Apelin-12	Vaspin	Change in Vaspin	Resistin	Change in Resistin
BMI (kg/m^2^)	−0.44 (*p* > 0.05)	−0.001 (*p* > 0.05)	−0.56 (*p* > 0.05)	0.53 (*p* > 0.05)	−0.93 * (*p* < 0.05)	0.53 (*p* > 0.05)
BMI z score	−0.26 (*p* > 0.05)	−0.19 (*p* > 0.05)	−0.68 (*p* > 0.05)	0.62 (*p* > 0.05)	−0.89 * (*p* < 0.05)	0.47 (*p* > 0.05)
TMI (kg/m^3^)	−0.27 (*p* > 0.05)	−0.09 (*p* > 0.05)	−0.62 (*p* > 0.05)	0.59 (*p* > 0.05)	−0.91 * (*p* < 0.05)	0.39 (*p* > 0.05)
WC (cm)	−0.44 (*p* > 0.05)	−0.19 (*p* > 0.05)	0.18 (*p* > 0.05)	−0.24 (*p* > 0.05)	−0.83 (*p* > 0.05)	0.93 * (*p* < 0.05)
WC z score	−0.29 (*p* > 0.05)	−0.36 (*p* > 0.05)	0.15 (*p* > 0.05)	−0.22 (*p* > 0.05)	−0.87 (*p* > 0.05)	0.93 * (*p* < 0.05)
HP (cm)	−0.50 (*p* > 0.05)	−0.10 (*p* > 0.05)	−0.54 (*p* > 0.05)	0.46 (*p* > 0.05)	−0.88 * (*p* < 0.05)	0.72 (*p* > 0.05)
WHtR	−0.38 (*p* > 0.05)	−0.24 (*p* > 0.05)	0.12 (*p* > 0.05)	−0.18 (*p* > 0.05)	−0.89 * (*p* < 0.05)	0.90 * (*p* < 0.05)
SBP (mmHg)	−0.64 (*p* > 0.05)	0.05 (*p* > 0.05)	0.16 (*p* > 0.05)	−0.25 (*p* > 0.05)	−0.37 (*p* > 0.05)	0.89 * (*p* < 0.05)
HbA1C (%)	−0.30 (*p* > 0.05)	−0.30 (*p* > 0.05)	0.33 (*p* > 0.05)	−0.40 (*p* > 0.05)	−0.77 (*p* > 0.05)	0.91 * (*p* < 0.05)
SGOT (mg/dL)	0.37 (*p* > 0.05)	0.25 (*p* > 0.05)	−0.39 (*p* > 0.05)	0.82 (*p* > 0.05)	0.40 (*p* > 0.05)	−0.93 * (*p* < 0.05)
ApoB (mg/dL)	−0.49 (*p* > 0.05)	0.17 (*p* > 0.05)	−0.88 * (*p* < 0.05)	0.84 (*p* > 0.05)	−0.67 (*p* > 0.05)	0.28 (*p* > 0.05)
Cortisol (μg/mL)	−0.92 * (*p* < 0.05)	0.85 (*p* > 0.05)	−0.39 (*p* > 0.05)	0.43 (*p* > 0.05)	−0.24 (*p* > 0.05)	0.18 (*p* > 0.05)
Muscle mass percentage (%)	−0.63 (*p* > 0.05)	0.08 (*p* > 0.05)	0.33 (*p* > 0.05)	−0.38 (*p* > 0.05)	−0.57 (*p* > 0.05)	0.91 * (*p* < 0.05)
Fat-free mass (kg)	−0.63 (*p* > 0.05)	0.08 (*p* > 0.05)	0.33 (*p* > 0.05)	−0.38 (*p* > 0.05)	−0.57 (*p* > 0.05)	0.91 * (*p* < 0.05)
TBW (kg)	−0.63 (*p* > 0.05)	0.08 (*p* > 0.05)	0.33 (*p* > 0.05)	−0.38 (*p* > 0.05)	−0.57 (*p* > 0.05)	0.90 * (*p* < 0.05)

Abbreviations: ApoB, apolipoprotein B; BMI, body mass index; HbA1C, hemoglobin A1c; HP, hip circumference; SBP, systolic blood pressure; SGOT, serum glutamic-oxaloacetic transaminase; TBW, total body water; TMI, tri-ponderal mass index; WC, waist circumference; WHtR, waist-to-height ratio. Correlations of the variables are evaluated by Pearson’s R coefficient. Statistical significance was set at *p* < 0.05. NS: nonsignificant (*p* > 0.05) difference. Statistically significant associations are shown in bold and indicated by asterisks.

**Table 4:** Correlation coefficients of the apelin-12, vaspin and resistin concentrations in subjects with obesity at initial assessment.

	Apelin-12	Change in Apelin-12	Vaspin	Change in Vaspin	Resistin	Change in Resistin
Age (years)	−0.10 (*p* > 0.05)	0.10 (*p* > 0.05)	−0.17 (*p* > 0.05)	−0.37 * (*p* < 0.05)	0.05 (*p* > 0.05)	−0.06 (*p* > 0.05)
BW (kg)	−0.09 (*p* > 0.05)	0.08 (*p* > 0.05)	−0.18 (*p* > 0.05)	−0.38 * (*p* < 0.05)	0.09 (*p* > 0.05)	−0.04 (*p* > 0.05)
Height (cm)	−0.02 (*p* > 0.05)	−0.002 (*p* > 0.05)	−0.16 (*p* > 0.05)	−0.31 * (*p* < 0.05)	0.06 (*p* > 0.05)	−0.08 (*p* > 0.05)
BMI (kg/m^2^)	−0.11 (*p* > 0.05)	0.10 (*p* > 0.05)	−0.18 (*p* > 0.05)	−0.33 * (*p* < 0.05)	0.11 (*p* > 0.05)	0.01 (*p* > 0.05)
Total cholesterol (mg/dL)	−0.07 (*p* > 0.05)	0.04 (*p* > 0.05)	0.19 (*p* > 0.05)	−0.30 * (*p* < 0.05)	−0.06 (*p* > 0.05)	0.12 (*p* > 0.05)
Apo-A1 (mg/dL)	−0.10 (*p* > 0.05)	0.11 (*p* > 0.05)	0.13 (*p* > 0.05)	−0.40 * (*p* < 0.05)	−0.09 (*p* > 0.05)	0.03 (*p* > 0.05)
Apo-B (mg/dL)	−0.04 (*p* > 0.05)	0.01 (*p* > 0.05)	0.13 (*p* > 0.05)	−0.40 (*p* < 0.05)	−0.12 (*p* > 0.05)	0.28 * (*p* < 0.05)
SGOT (mg/dL)	0.14 (*p* > 0.05)	−0.13 (*p* > 0.05)	−0.08 (*p* > 0.05)	−0.14 (*p* > 0.05)	−0.25 * (*p* < 0.05)	0.18 (*p* > 0.05)
HsCRP (mg/L)	−0.17 (*p* > 0.05)	0.16 (*p* > 0.05)	−0.03 (*p* > 0.05)	−0.43 * (*p* < 0.05)	0.17 (*p* > 0.05)	0.02 (*p* > 0.05)
Cortisol (μg/mL)	−0.18 (*p* > 0.05)	0.18 (*p* > 0.05)	−0.11 (*p* > 0.05)	−0.43 * (*p* < 0.05)	0.20 (*p* > 0.05)	−0.09 (*p* > 0.05)
Vitamin D (ng/mL)	0.10 (*p* > 0.05)	−0.11 (*p* > 0.05)	0.64 * (*p* < 0.05)	−0.17 (*p* > 0.05)	−0.06 (*p* > 0.05)	0.07 (*p* > 0.05)
Fat mass (kg)	−0.21 (*p* > 0.05)	0.19 (*p* > 0.05)	0.37 * (*p* < 0.05)	−0.42 * (*p* < 0.05)	0.13 (*p* > 0.05)	−0.01 (*p* > 0.05)
Muscle mass percentage (%)	−0.13 (*p* > 0.05)	0.10 (*p* > 0.05)	0.28 (*p* > 0.05)	−0.33 * (*p* < 0.05)	0.04 (*p* > 0.05)	0.06 (*p* > 0.05)
Bone mass (kg)	−0.15 (*p* > 0.05)	0.12 (*p* > 0.05)	0.28 (*p* > 0.05)	−0.33 * (*p* < 0.05)	0.03 (*p* > 0.05)	0.01 (*p* > 0.05)
Fat-free mass (kg)	−0.13 (*p* > 0.05)	0.11 (*p* > 0.05)	0.28 (*p* > 0.05)	−0.33 * (*p* < 0.05)	0.04 (*p* > 0.05)	0.06 (*p* > 0.05)
TBW (kg)	−0.13 (*p* > 0.05)	0.11 (*p* > 0.05)	0.30 (*p* > 0.05)	−0.34 * (*p* < 0.05)	0.04 (*p* > 0.05)	0.02 (*p* > 0.05)
Adiponectin (ng/mL)	0.14 (*p* > 0.05)	−0.16 (*p* > 0.05)	0.29 * (*p* < 0.05)	−0.05 (*p* > 0.05)	0.15 (*p* > 0.05)	−0.12 (*p* > 0.05)
Leptin (ng/mL)	0.05 (*p* > 0.05)	−0.06 (*p* > 0.05)	−0.09 (*p* > 0.05)	−0.39 * (*p* < 0.05)	0.18 (*p* > 0.05)	−0.08 (*p* > 0.05)
Osteopontin (ng/mL)	0.37 * (*p* < 0.05)	−0.32 * (*p* < 0.05)	−0.14 (*p* > 0.05)	0.03 (*p* > 0.05)	0.05 (*p* > 0.05)	−0.09 (*p* > 0.05)
Apelin-12 (ng/mL)	NA	ΝA	−0.01 (*p* > 0.05)	−0.07 (*p* > 0.05)	−0.19 (*p* > 0.05)	0.13 (*p* > 0.05)
Change in apelin-12	ΝA	NA	0.06 (*p* > 0.05)	0.07 (*p* > 0.05)	0.14 (*p* > 0.05)	−0.10 (*p* > 0.05)
Vaspin (pg/mL)	−0.09 (*p* > 0.05)	0.006 (*p* > 0.05)	NA	NA	−0.04 (*p* > 0.05)	0.08 (*p* > 0.05)
Change in vaspin	−0.07 (*p* > 0.05)	0.07 (*p* > 0.05)	NA	NA	−0.04 (*p* > 0.05)	0.07 (*p* > 0.05)
Resistin (ng/mL)	−0.19 (*p* > 0.05)	0.15 (*p* > 0.05)	−0.04 (*p* > 0.05)	−0.17 (*p* > 0.05)	NA	NA
Change in resistin	−0.10 (*p* > 0.05)	−0.10 (*p* > 0.05)	0.08 (*p* > 0.05)	0.07 (*p* > 0.05)	NA	NA

Abbreviations: ApoA1, apolipoprotein A1; ApoB, apolipoprotein B; BMI, body mass index; BW, body weight; hsCRP, high-sensitivity C-reactive protein; SGOT, serum glutamic-oxaloacetic transaminase; TBW, total body water; 25-OH-Vitamin D, total 25-OH vitamin D. Correlations of the variables are evaluated by Pearson’s R coefficient. Statistical significance was set at *p* < 0.05. NA: Not applicable; NS: nonsignificant (*p* > 0.05) difference. Statistically significant associations are shown in bold and indicated by asterisks.

**Table 5:** Correlation coefficient of the apelin-12, vaspin and resistin concentrations in subjects with overweight at initial assessment.

	Apelin-12	Change in Apelin-12	Vaspin	Change in Vaspin	Resistin	Change in Resistin
WHR	0.04 (*p* > 0.05)	0.0006 (*p* > 0.05)	0.54 * (*p* < 0.05)	−0.20 (*p* > 0.05)	0.13 (*p* > 0.05)	0.11 (*p* > 0.05)
Glucose (mg/dL)	0.03 (*p* > 0.05)	−0.06 (*p* > 0.05)	0.55 * (*p* < 0.05)	−0.54 * (*p* < 0.05)	−0.10 (*p* > 0.05)	0.12 (*p* > 0.05)
HOMA-IR	−0.06 (*p* < 0.05)	0.06 (*p* < 0.05)	0.27 (*p* < 0.05)	−0.49 * (*p* < 0.05)	0.12 (*p* < 0.05)	−0.05 (*p* < 0.05)
LDL (mg/dL)	0.05 (*p* > 0.05)	−0.07 (*p* > 0.05)	−0.53 * (*p* < 0.05)	0.45 (*p* > 0.05)	−0.07 (*p* > 0.05)	−0.26 (*p* > 0.05)
Albumin (g/dL)	−0.22 (*p* > 0.05)	0.13 (*p* > 0.05)	−0.49 * (*p* < 0.05)	0.30 (*p* > 0.05)	−0.11 (*p* > 0.05)	0.02 (*p* > 0.05)
γGT (mg/dL)	0.45 * (*p* < 0.05)	−0.42 (*p* > 0.05)	−0.13 (*p* > 0.05)	0.13 (*p* > 0.05)	0.08 (*p* > 0.05)	0.27 (*p* > 0.05)
Cortisol (μg/dL)	0.40 (*p* > 0.05)	−0.50 * (*p* < 0.05)	−0.02 (*p* > 0.05)	−0.01 (*p* > 0.05)	0.08 (*p* > 0.05)	−0.14 (*p* > 0.05)
Vitamin D (ng/mL)	−0.11 (*p* > 0.05)	−0.04 (*p* > 0.05)	0.14 (*p* > 0.05)	−0.12 (*p* > 0.05)	−0.34 * (*p* < 0.05)	0.25 (*p* > 0.05)
Fat mass (kg)	−0.35 (*p* > 0.05)	0.41 (*p* > 0.05)	0.19 (*p* > 0.05)	−0.15 (*p* > 0.05)	0.40 * (*p* < 0.05)	−0.33 (*p* > 0.05)
Leptin (ng/mL)	−0.18 (*p* > 0.05)	0.15 (*p* > 0.05)	−0.25 (*p* > 0.05)	0.24 (*p* > 0.05)	0.40 * (*p* < 0.05)	−0.14 (*p* > 0.05)
FGF-21 (pg/mL)	0.55 * (*p* < 0.05)	−0.50 * (*p* < 0.05)	0.25 (*p* > 0.05)	−0.24 (*p* > 0.05)	0.05 (*p* > 0.05)	0.02 (*p* > 0.05)
Sclerostin (pg/mL)	−0.36 (*p* > 0.05)	0.39 (*p* > 0.05)	0.09 (*p* > 0.05)	−0.10 (*p* > 0.05)	0.54 * (*p* < 0.05)	−0.40 (*p* > 0.05)
Apelin-12 (ng/mL)	NA	NA	−0.13 (*p* > 0.05)	0.19 (*p* > 0.05)	−0.20 (*p* > 0.05)	0.17 (*p* > 0.05)
Change in apelin-12	NA	NA	0.15 (*p* > 0.05)	−0.19 (*p* > 0.05)	0.28 (*p* > 0.05)	−0.24 (*p* > 0.05)
Vaspin (pg/mL)	−0.13 (*p* > 0.05)	0.15 (*p* > 0.05)	NA	−0.19 (*p* > 0.05)	0.16 (*p* > 0.05)	0.12 (*p* > 0.05)
Change in vaspin	0.19 (*p* > 0.05)	−0.19 (*p* > 0.05)	NA	NA	0.16 (*p* > 0.05)	0.12 (*p* > 0.05)
Resistin (ng/mL)	−0.20 (*p* > 0.05)	0.28 (*p* > 0.05)	0.16 (*p* > 0.05)	0.16 (*p* > 0.05)	NA	NA
Change in resistin	0.17 (*p* > 0.05)	−0.24 (*p* > 0.05)	0.12 (*p* > 0.05)	0.12 (*p* > 0.05)	NA	NA

Abbreviations: FGF-21, fibroblast growth factor-21; HOMA-IR, homeostatic model assessment for insulin resistance; γGT, gamma-glutamyl transferase; LDL, low-density lipoprotein; Vitamin D, total 25-OH vitamin D; WHR, waist-to-hip ratio. Correlations of the studied variables are evaluated by Pearson’s R coefficient. Statistical significance was set at *p* < 0.05. NA: not applicable; NS: nonsignificant (*p* > 0.05) difference. Statistically significant associations are shown in bold and indicated by asterisks.

**Table 6:** Correlation coefficient of the apelin-12, vaspin and resistin concentrations in prepubertal subjects at initial assessment.

	Apelin-12	Change in Apelin-12	Vaspin	Change in Vaspin	Resistin	Change in Resistin
BMI (kg/m^2^)	−0.03 (*p* > 0.05)	−0.04 (*p* > 0.05)	−0.14 (*p* > 0.05)	0.22 (*p* > 0.05)	0.29 * (*p* < 0.05)	−0.17 (*p* > 0.05)
TMI (kg/m^3^)	−0.14 (*p* > 0.05)	0.13 (*p* > 0.05)	−0.11 (*p* > 0.05)	0.40 * (*p* < 0.05)	0.12 (*p* > 0.05)	−0.02 (*p* > 0.05)
LDL (mg/dL)	−0.13 (*p* > 0.05)	0.08 (*p* > 0.05)	0.16 (*p* > 0.05)	0.37 * (*p* < 0.05)	−0.09 (*p* > 0.05)	−0.08 (*p* > 0.05)
Vitamin D (ng/mL)	−0.03 (*p* > 0.05)	0.03 (*p* > 0.05)	0.78 * (*p* < 0.05)	−0.31 (*p* > 0.05)	0.12 (*p* > 0.05)	0.06 (*p* > 0.05)
Cortisol (μg/dL)	0.51 * (*p* < 0.05)	−0.51 * (*p* < 0.05)	−0.12 (*p* > 0.05)	0.09 (*p* > 0.05)	0.11 (*p* > 0.05)	−0.16 (*p* > 0.05)
Apelin-12 (ng/mL)	NA	NA	−0.05 (*p* > 0.05)	−0.10 (*p* > 0.05)	−0.04 (*p* > 0.05)	0.05 (*p* > 0.05)
Change in apelin-12	NA	NA	0.05 (*p* > 0.05)	0.08 (*p* > 0.05)	0.004 (*p* > 0.05)	−0.04 (*p* > 0.05)
Vaspin (pg/mL)	−0.05 (*p* > 0.05)	0.05 (*p* > 0.05)	NA	NA	0.07 (*p* > 0.05)	0.09 (*p* > 0.05)
Change in vaspin	−0.10 (*p* > 0.05)	0.08 (*p* > 0.05)	NA	NA	−0.24 (*p* > 0.05)	−0.10 (*p* > 0.05)
Resistin (ng/mL)	−0.04 (*p* > 0.05)	0.004 (*p* > 0.05)	0.07 (*p* > 0.05)	−0.24 (*p* > 0.05)	NA	NA
Change in resistin	0.05 (*p* > 0.05)	−0.04 (*p* > 0.05)	0.09 (*p* > 0.05)	−0.10 (*p* > 0.05)	NA	NA
Irisin (ng/mL)	−0.07 (*p* > 0.05)	−0.05 (*p* > 0.05)	−0.08 (*p* > 0.05)	−0.02 (*p* > 0.05)	0.47 * (*p* < 0.05)	−0.44 * (*p* < 0.05)
FGF-21 (pg/mL)	0.76 * (*p* < 0.05)	−0.75 * (*p* < 0.05)	−0.15 (*p* > 0.05)	−0.13 (*p* > 0.05)	0.22 (*p* > 0.05)	−0.07 (*p* > 0.05)
FGF-23 (pg/mL)	−0.09 (*p* > 0.05)	0.09 (*p* > 0.05)	−0.08 (*p* > 0.05)	0.02 (*p* > 0.05)	0.30 * (*p* < 0.05)	−0.27 (*p* > 0.05)

Abbreviations: BMI, body mass index; FGF, fibroblast growth factor; LDL, low-density lipoprotein; TMI, tri-ponderal mass index; Vitamin D, total 25-OH vitamin D. Correlations of the variables are evaluated by Pearson’s R coefficient. Statistical significance was set at *p* < 0.05. NA: not applicable; NS: nonsignificant (*p* > 0.05) difference. Statistically significant associations are shown in bold and indicated by asterisks.

**Table 7:** Correlation coefficient of the apelin-12, vaspin and resistin concentrations in pubertal subjects at initial assessment.

	Apelin-12	Change in Apelin-12	Vaspin	Change in Vaspin	Resistin	Change in Resistin
Ca (mmol/L)	0.33 * (*p* < 0.05)	−0.30 (*p* > 0.05)	0.24 (*p* > 0.05)	−0.22 (*p* > 0.05)	0.04 (*p* > 0.05)	−0.16 (*p* > 0.05)
HsCRP	−0.18 (*p* > 0.05)	0.17 (*p* > 0.05)	0.34 * (*p* < 0.05)	−0.42 * (*p* < 0.05)	0.20 (*p* > 0.05)	−0.06 (*p* > 0.05)
Glucose (mg/dL)	0.43 * (*p* < 0.05 **)**	−0.33 (*p* > 0.05)	0.006 (*p* > 0.05)	−0.02 (*p* > 0.05)	−0.33 * (*p* < 0.05)	0.31 * (*p* < 0.05)
Total cholesterol (mg/dL)	0.06 (*p* > 0.05)	−0.10 (*p* > 0.05)	0.34 * (*p* < 0.05)	−0.40 * (*p* < 0.05)	−0.08 (*p* > 0.05)	0.04 (*p* > 0.05)
HDL (mg/dL)	−0.1 (*p* > 0.05)	0.07 (*p* > 0.05)	0.33 * (*p* < 0.05)	−0.36 * (*p* < 0.05)	−0.01 (*p* > 0.05)	0.06 (*p* > 0.05)
ApoA1 (mg/dL)	−0.09 (*p* > 0.05)	0.08 (*p* > 0.05)	0.33 * (*p* < 0.05)	−0.39 * (*p* < 0.05)	0.02 (*p* > 0.05)	0.02 (*p* > 0.05)
Vitamin D (ng/mL)	0.16 (*p* > 0.05)	−0.27 (*p* > 0.05)	0.06 (*p* > 0.05)	−0.06 (*p* > 0.05)	−0.31 * (*p* < 0.05)	0.16 (*p* > 0.05)
Cortisol (μg/dL)	−0.33 * (*p* < 0.05)	0.31 (*p* > 0.05)	0.42 * (*p* < 0.05)	−0.46 * (*p* < 0.05)	0.09 (*p* > 0.05)	0.01 (*p* > 0.05)
Osteopontin (ng/mL)	0.40 * (*p* < 0.05)	−0.35 * (*p* < 0.05)	0.04 (*p* > 0.05)	0.02 (*p* > 0.05)	−0.12 (*p* > 0.05)	0.07 (*p* > 0.05)
Apelin-12 (ng/mL)	NA	NA	−0.06 (*p* > 0.05)	0.08 (*p* > 0.05)	−0.26 (*p* > 0.05)	0.14 (*p* > 0.05)
Change in apelin-12	NA	NA	0.04 (*p* > 0.05)	−0.05 (*p* > 0.05)	0.27 (*p* > 0.05)	−0.15 (*p* > 0.05)
Vaspin (pg/mL)	−0.06 (*p* > 0.05)	0.04 (*p* > 0.05)	NA	NA	−0.03 (*p* > 0.05)	0.03 (*p* > 0.05)
Change in vaspin	0.08 (*p* > 0.05)	−0.05 (*p* > 0.05)	NA	NA	−0.06 (*p* > 0.05)	0.005 (*p* > 0.05)
Resistin (ng/mL)	−0.26 (*p* > 0.05)	0.27 (*p* > 0.05)	−0.03 (*p* > 0.05)	−0.06 (*p* > 0.05)	NA	NA
Change in resistin	0.14 (*p* > 0.05)	−0.15 (*p* > 0.05)	0.03 (*p* > 0.05)	0.005 (*p* > 0.05)	NA	NA

Abbreviations: Apo-A1, apolipoprotein A1; HDL, high-density lipoprotein; hsCRP, high-sensitivity C-reactive protein; Vitamin D, total 25-OH vitamin D. Correlations of the variables are evaluated by Pearson’s R coefficient. Statistical significance was set at *p* < 0.05. NA: not applicable; NS: nonsignificant (*p* > 0.05) difference. Statistically significant associations are shown in bold and indicated by asterisks.

## Data Availability

The data presented in this study are available upon request from the corresponding author. The data are not publicly available due to privacy restrictions.

## Supplementary Materials

The following supporting information can be downloaded at: https://www.mdpi.com/article/10.3390/nu16213646/s1, Table S1: Assessed variables of all subjects at initial assessment and at annual assessment. Subjects were classified as obese and overweight according to IOTF criteria at initial assessment. The respective statistically significant differences between the two groups (as classified at initial assessment) are presented; Table S2: Standard forward, stepwise multiple regression of anthropometric parameters; Table S3: Standard forward, stepwise multiple regression of metabolic syndrome parameters; Table S4: Standard forward, stepwise multiple regression of glucose metabolism parameters; Table S5: Standard forward, stepwise multiple regression of adiposity parameters; Table S6: Standard forward, stepwise multiple regression of bone metabolism parameters.
