# Supplementary material for: The Effect of a Multidisciplinary Lifestyle Intervention Program on Apelin-12, Vaspin and Resistin Concentrations in Children and Adolescents with Overweight and Obesity
Source: Nutrients. 2024 Oct 26;16(21):3646. doi: 10.3390/nu16213646 (PMC11547676; doi:10.3390/nu16213646)
Supplement: Supplementary file 1 [file nutrients-16-03646-s001.zip › nutrients-3207574-supplementary.pdf]

SUPPLEMENTAL TABLES

Table S1. Assessed variables of all subjects at initial assessment and at annual assessment. Subjects were classified as obese and overweight according to IOTF criteria at initial assessment. The respective statistically significant differences between the two groups (as classified at initial assessment) are presented.

	**Initial assessment**			**Annual assessment**			
	**Obese**	**Overweight**	**P_{within}**	**Obese**	**Overweight**	**P_{within}**	**$P_{between\ timepoints}$**
Adiponectin	20.755,93 (±1.908,78)	26.258,03 (±3.783,20)	NS	21.452,66 (±2.132,18)	24.849,32 (±3.054,27)	NS	NS/NS
Leptin	34,05 (±3,09)	23,48 (±3,21)	**<0.01****	22,43 (±2,11)	17,09 (±2,22)	NS	NS/**<0.01****
Apelin-12	245,84 (±38,82)	280,55 (±71,31)	NS	56,07 (±6,84)	60,20 (±7,25)	NS	**<0.01****/**<0.01****
Vaspin	0,24 (±3,21)	0,19 (±0,10)	NS	0,32 (±0,10)	0,24 (±0,02)	NS	**<0.01****/**<0.01****
Resistin	3.910,51 (±244,99)	3.408,26 (±229,50)	NS	3.059,13 (±148,62)	2.822,65 (±227,74)	NS	NS/**<0.01****

Abbreviations: IOTF, international obesity task force. All variables are presented as mean ± SE of mean; All measured variables were compared by employing one-way ANOVA; Significant main effects were revealed by the LSD post-hoc test; Statistical significance was set at $p < 0.05$, as shown in bold and indicated by an asterisk, while strong significance was set at $p < 0.01$, as shown in bold and indicated by two asterisks; NS: nonsignificant (p > 0.05)

Table S2: Standard forward, stepwise multiple regression of anthropometric parameters.

	**BMI z score**	**Body Weight**	**Height**	**TMI**
Resistin_t12	**b=0.437, p<0.05***	NS	NS	NS
Change in vaspin	**b= -0.794, p<0.05***	**b= -2.556, p<0.05***	**b=2.401, p<0.05***	**b=2.132, p<0.05***

Abbreviations: resistin_t12, resistin concentrations at annual assessment; BMI, body mass index, TMI, tri-ponderal mass index; variables are presented as b-values; measurements were taken as independent variables: body weight, height, BMI, BMI z score, TMI, waist and hip circumference, waist-to-hip ratio, and waist-to-height ratio at initial assessment; Statistical significance was set at $p < 0.05$, as shown in bold and indicated by an asterisk; NS: nonsignificant (p > 0.05) difference.

Table S3: Standard forward, stepwise multiple regression of metabolic syndrome parameters.

	**Glucose**	**HDL**
Apelin_t12	**b= 0.236, p < 0.05***	NS
Change in vaspin	**b= -0.281, p < 0.05***	**b= -0.284, p < 0.05***

Abbreviations: apelin_t12, apelin concentrations at annual assessment; HDL, high density lipoprotein; variables are presented as b-values; measurements were taken as independent variables: glucose, systolic blood pressure, waist circumference, triglycerides and HDL at initial assessment; Statistical significance was set at $p < 0.05$, as shown in bold and indicated by an asterisk; NS: nonsignificant ($p > 0.05$) difference.

Table S4: Standard forward, stepwise multiple regression of glucose metabolism parameters.

	**Glucose**	**HbA1c**
Apelin_t12	**b= 0.328, p < 0.05***	NS
Change in vaspin	NS	**b= -0.262, p < 0.05***

Abbreviations: apelin_t12, apelin concentrations at annual assessment; HbA1C, hemoglobin A1c; variables are presented as b-values; measurements were taken as independent variables: glucose, insulin, HbA1C and homeostatic model assessment for insulin resistance at initial assessment; Statistical significance was set at $p < 0.05$, as shown in bold and indicated by an asterisk; NS: nonsignificant ($p > 0.05$) difference.

Table S5: Standard forward, stepwise multiple regression of adiposity parameters.

	**Waist z score**
Change in apelin	**b= -0.671, p < 0.05***

Variables are presented as b-values; measurements were taken as independent variables: adiponectin and leptin concentrations, waist circumference, waist circumference z score, waist-to-height ratio and fat mass at initial assessment; Statistical significance was set at $p < 0.05$, as shown in bold and indicated by an asterisk; NS: nonsignificant ($p > 0.05$) difference.

Table S6: Standard forward, stepwise multiple regression of bone metabolism parameters.

	**Osteopontin**	**Vitamin D**
Change in apelin	**b= -0.299, p < 0.05***	NS
Vaspin_t12	NS	**b= 0.621, p < 0.05***

Abbreviations: vaspin_12, vaspin concentrations at annual assessment; vitamin D, total 25-OH-vitamin D. Variables are presented as b-values; measurements were taken as independent variables: calcium, phosphorus, alkaline phosphatase, parathormone, vitamin D, osteopontin and fibroblast growth factor-23 at initial assessment; Statistical significance was set at $p < 0.05$, as shown in bold and indicated by an asterisk; NS: nonsignificant ($p > 0.05$) difference.
